# Tobacco-Related Clinical Services and Tobacco-Free Policies in Behavioral Health Treatment Facilities — United States, 2023

**DOI:** 10.15585/mmwr.mm7414a3

**Published:** 2025-04-24

**Authors:** Brenna VanFrank, Emilia Pasalic, Briana Oliver, Kevin Caron, Karuna Nerurkar, Kristy Marynak, Ahmed Jamal, Krishna Palipudi, Margaret Mahoney, Anna Schecter, Chanel Recasner, Elizabeth Hazelwood, Naomi Tomoyasu

**Affiliations:** ^1^Office on Smoking and Health, National Center for Chronic Disease Prevention and Health Promotion, CDC; ^2^Center for Behavioral Health Statistics and Quality, Substance Abuse and Mental Health Services Administration, Rockville, Maryland; ^3^Katmai Government Services, LLC., Orlando, Florida; ^4^Division of Overdose Prevention, National Center for Injury Prevention and Control, CDC.

**Summary**

**What is already known about this topic?**

Incorporation of tobacco cessation treatments and tobacco-free policies into substance use and mental health treatment facilities could help decrease tobacco use among persons with behavioral health conditions.

**What is added by this report?**

In 2023, counseling was the most commonly offered tobacco cessation service in mental health (53.1%) and substance use (69.9%) treatment facilities. Fewer than one half of facilities offered tobacco cessation pharmacotherapy. Tobacco-free policies were reported by 53.9% of mental health facilities and 33.9% of substance use facilities.

**What are the implications for public health practice?**

Integrating tobacco treatment services into behavioral health care and making treatment settings tobacco-free could support cessation and help decrease tobacco-related disease, and might improve behavioral health outcomes.

## Abstract

Evidence-based cessation treatments and tobacco-free policies support and increase smoking cessation, which has positive physical health impacts and is associated with positive behavioral health outcomes. Implementation of these strategies in substance use and mental health treatment facilities (behavioral health treatment facilities) could help decrease tobacco use among persons with behavioral health conditions. Data from the 2023 National Substance Use and Mental Health Services Survey were analyzed to ascertain the number and percentage of behavioral health treatment facilities that offered tobacco-related clinical services and had tobacco-free policies. In 2023, tobacco cessation counseling was the most commonly offered cessation service in facilities treating mental health conditions (53.1%) and substance use disorders (69.9%). Fewer than one half of facilities offered nicotine replacement therapy (35.0% of mental health and 40.2% of substance use facilities) or non-nicotine cessation medication (33.6% of mental health and 35.3% of substance use facilities). Policies prohibiting both smoking and vaping were reported by 53.9% of mental health and 33.9% of substance use facilities. Among facilities with a tobacco-free policy, 64.4% of mental health and 81.8% of substance use facilities offered at least one cessation service. Opportunities remain to improve cessation supports in behavioral health treatment facilities, including tobacco-free policies and integrated tobacco cessation treatment services. These strategies could help decrease tobacco-related disease and improve behavioral health outcomes.

## Introduction

Persons with mental health conditions or substance use disorders (i.e., behavioral health conditions) have a disproportionately high prevalence of commercial tobacco product* use and are more likely to experience smoking-related illness than are those without such conditions (*1*–*3*). Cigarette smoking causes numerous diseases and is associated with negative behavioral health outcomes (*3*). Quitting smoking has substantial positive physical health impacts and is associated with positive behavioral health outcomes (*3*–*7*).

Smoking cessation can be supported and increased through provision and use of evidence-based treatments (behavioral counseling and pharmacotherapy) and implementation of smoke-free policies (*4*). Pairing smoke-free policies with the availability of tobacco cessation treatment might further support cessation (*4*). However, access to these types of cessation supports in behavioral health treatment settings has been limited. In 2016, fewer than one half of U.S. substance use and mental health treatment facilities provided tobacco cessation treatments or had policies prohibiting smoking in all indoor and outdoor areas (*8*). This might be partly explained by the historical normalization of smoking in behavioral health settings, tobacco industry influence (e.g., industry provision of reduced-cost or free cigarettes to treatment facilities), and persistence of misinformation regarding the potential of smoking cessation to negatively impact behavioral health outcomes (*3*,*5*,*9*,*10*). To update and expand upon previous estimates of cessation supports in substance use and mental health treatment facilities in the United States, CDC and the Substance Abuse Mental Health Service Administration (SAMHSA) analyzed data from the 2023 National Substance Use and Mental Health Services Survey (N-SUMHSS).

## Methods

### Data Source

SAMHSA conducts N-SUMHSS annually among all facilities that provide mental health or substance use treatment services across 50 states, seven territories, and the District of Columbia (DC).^†^ The overall response rate for the 2023 N-SUMHSS was 85%. Estimates for substance use and mental health facilities are not mutually exclusive because some facilities offer both types of services.

### Analysis

The number and percentage of facilities that offer tobacco-related clinical services and that have tobacco-free policies were assessed. Tobacco-related clinical services included tobacco use screening, tobacco cessation counseling, nicotine replacement therapy (NRT), and non-nicotine cessation medications (bupropion and varenicline). Tobacco-free policies prohibit both smoking (smoke-free policies) and vaping (vape-free policies) in all facility indoor and outdoor areas.

Results were stratified by jurisdiction (50 states, DC, and Puerto Rico), facility operation (private for-profit, private nonprofit, and public agency), and service setting (24-hour hospital inpatient, outpatient, partial hospitalization or day treatment, and 24-hour residential for mental health facilities; hospital inpatient, outpatient, and residential for substance use facilities).^§^ In addition, the percentage of facilities that provided at least one cessation service (counseling, NRT, or non-nicotine medications) among facilities with tobacco-free policies was assessed nationally and by jurisdiction. Analyses were conducted using Python (version 3.12.2; Python Software Foundation). Facilities with missing data were excluded from prevalence calculations only for the measures for which data were missing. This activity was reviewed by SAMHSA, deemed not research, and was conducted consistent with applicable federal law^¶^; CDC deferred to SAMHSA’s determination.

## Results

### Tobacco-Related Clinical Services

The study sample included 9,856 mental health facilities and 14,620 substance use facilities. Tobacco use screening was offered at 6,796 (69.2%) mental health and 11,978 (82.3%) substance use facilities (Table). Tobacco cessation counseling was the most commonly offered cessation service (53.1% of mental health and 69.9% of substance use facilities). Fewer than one half of facilities offered NRT (35.0% of mental health and 40.2% of substance use facilities) or non-nicotine cessation medications (33.6% of mental health and 35.3% of substance use facilities). The highest percentages of facilities offering tobacco-related clinical services were public agency–operated facilities (when stratified by facility operation) and hospital inpatient facilities (when stratified by service setting), irrespective of facility type or service.

**TABLE:** Percentage and number of behavioral health treatment facilities that offer tobacco screening or cessation treatment, or that prohibit smoking or vaping in all indoor and outdoor areas, by facility type* — National Substance Use and Mental Health Services Survey, United States, 2023

Facility type/Characteristic/Jurisdiction	Facilities, no.	Facilities with tobacco cessation services offered or tobacco-free policies in place, % (no.)						
		Tobacco use screening^†^	Tobacco cessation counseling^§^	Nicotine replacement therapy^¶^	Non-nicotine cessation medications**	Smoke-free policy^††^	Vape-free policy^§§^	Tobacco-free policy^¶¶^
**Mental health treatment facilities**								
**Overall**	**9,856**	**69.2 (6,796)**	**53.1 (5,212)**	**35.0 (3,439)**	**33.6 (3,301)**	**54.6 (5,374)**	**57.9 (5,694)**	**53.9 (5,306)**
**Facility operation**								
Private for-profit	2,295	53.2 (1,216)	45.6 (1,041)	29.2 (667)	26.8 (611)	37.7 (865)	43.8 (1,001)	37.1 (849)
Private nonprofit	5,876	70.7 (4,148)	50.3 (2,948)	32.6 (1,909)	31.5 (1,849)	56.1 (3,298)	58.7 (3,449)	55.4 (3,253)
Public agency or department	1,675	85.5 (1,430)	73.0 (1,220)	51.5 (860)	50.2 (839)	72.2 (1,210)	74.2 (1,243)	71.8 (1,203)
Service setting***								
24-hr hospital inpatient	1,184	91.0 (1,074)	81.0 (956)	80.8 (954)	69.8 (824)	84.8 (1,003)	91.1 (1,077)	84.4 (998)
Outpatient	7,971	69.8 (5,560)	53.2 (4,231)	32.4 (2,579)	32.4 (2,575)	52.9 (4,214)	55.2 (4,396)	52.2 (4,155)
Partial hospitalization or day treatment	1,437	66.7 (957)	52.0 (745)	37.2 (533)	34.3 (491)	50.2 (721)	55.6 (799)	49.8 (716)
24-hr residential	1,611	58.3 (933)	44.7 (714)	32.6 (520)	29.2 (467)	51.9 (835)	58.6 (944)	51.1 (822)
**Jurisdiction**								
Alabama	126	70.6 (89)	46.8 (59)	35.7 (45)	33.3 (42)	45.2 (57)	48.4 (61)	45.2 (57)
Alaska	81	76.5 (62)	45.7 (37)	41.3 (33)	33.8 (27)	63.0 (51)	65.4 (53)	63.0 (51)
Arizona	361	66.1 (238)	54.2 (195)	30.4 (109)	34.5 (124)	31.4 (113)	36.7 (132)	30.8 (111)
Arkansas	150	58.7 (88)	56.0 (84)	32.7 (49)	28.7 (43)	42.0 (63)	44.0 (66)	40.0 (60)
California	804	50.4 (404)	33.1 (265)	21.1 (169)	18.1 (145)	47.8 (383)	51.9 (416)	47.2 (378)
Colorado	149	85.1 (126)	53.4 (79)	31.8 (47)	36.5 (54)	47.0 (70)	48.3 (72)	47.0 (70)
Connecticut	181	73.9 (133)	61.7 (111)	46.7 (84)	43.3 (78)	68.0 (123)	71.3 (129)	67.4 (122)
Delaware	35	72.7 (24)	45.5 (15)	42.4 (14)	39.4 (13)	54.3 (19)	57.1 (20)	54.3 (19)
District of Columbia	16	50.0 (8)	50.0 (8)	37.5 (6)	37.5 (6)	50.0 (8)	75.0 (12)	50.0 (8)
Florida	374	59.1 (220)	42.5 (158)	35.2 (131)	30.4 (113)	52.0 (194)	56.8 (212)	51.5 (192)
Georgia	178	79.2 (141)	62.9 (112)	34.8 (62)	33.7 (60)	52.8 (94)	55.6 (99)	52.2 (93)
Hawaii	19	84.2 (16)	78.9 (15)	52.6 (10)	73.7 (14)	42.1 (8)	63.2 (12)	42.1 (8)
Idaho	76	48.7 (37)	30.3 (23)	26.3 (20)	23.7 (18)	34.2 (26)	36.8 (28)	34.2 (26)
Illinois	317	59.2 (187)	38.0 (120)	29.7 (94)	28.5 (90)	49.5 (157)	55.5 (176)	49.2 (156)
Indiana	279	91.8 (256)	70.6 (197)	53.0 (148)	53.4 (149)	76.0 (212)	73.5 (205)	72.4 (202)
Iowa	113	67.0 (75)	35.7 (40)	22.3 (25)	25.0 (28)	54.0 (61)	55.8 (63)	53.1 (60)
Kansas	93	83.9 (78)	63.4 (59)	32.3 (30)	50.5 (47)	67.4 (62)	69.6 (64)	67.4 (62)
Kentucky	204	78.4 (160)	56.9 (116)	41.7 (85)	35.3 (72)	38.2 (78)	41.2 (84)	38.2 (78)
Louisiana	130	69.2 (90)	76.2 (99)	56.9 (74)	52.3 (68)	54.6 (71)	64.6 (84)	53.8 (70)
Maine	133	66.9 (89)	44.4 (59)	27.1 (36)	25.6 (34)	66.2 (88)	66.9 (89)	65.4 (87)
Maryland	218	59.6 (130)	45.9 (100)	32.1 (70)	27.1 (59)	43.6 (95)	46.3 (101)	42.2 (92)
Massachusetts	214	75.0 (159)	60.4 (128)	39.2 (83)	40.1 (85)	64.8 (138)	67.1 (143)	64.3 (137)
Michigan	303	73.9 (224)	66.0 (200)	40.3 (122)	39.3 (119)	58.4 (177)	67.0 (203)	58.4 (177)
Minnesota	229	72.4 (165)	49.6 (113)	45.4 (104)	34.2 (78)	50.0 (114)	52.2 (119)	50.0 (114)
Mississippi	135	59.7 (80)	42.5 (57)	21.6 (29)	15.7 (21)	38.5 (52)	43.0 (58)	38.5 (52)
Missouri	181	83.4 (151)	70.9 (127)	49.7 (89)	48.0 (86)	65.7 (119)	66.9 (121)	65.2 (118)
Montana	78	67.9 (53)	64.1 (50)	16.7 (13)	12.8 (10)	55.1 (43)	47.4 (37)	47.4 (37)
Nebraska	128	74.2 (95)	56.7 (72)	25.2 (32)	26.8 (34)	52.3 (67)	57.0 (73)	52.3 (67)
Nevada	77	61.8 (47)	40.8 (31)	26.3 (20)	26.3 (20)	29.9 (23)	36.4 (28)	29.9 (23)
New Hampshire	60	85.0 (51)	76.7 (46)	65.0 (39)	71.7 (43)	70.0 (42)	78.3 (47)	65.0 (39)
New Jersey	255	60.7 (153)	43.0 (108)	26.4 (66)	25.6 (64)	37.3 (95)	42.9 (109)	37.0 (94)
New Mexico	81	76.5 (62)	69.1 (56)	21.0 (17)	27.2 (22)	37.0 (30)	43.2 (35)	35.8 (29)
New York	565	91.7 (517)	78.7 (443)	55.8 (314)	57.5 (324)	76.2 (430)	77.7 (438)	75.9 (428)
North Carolina	235	60.4 (142)	52.8 (124)	33.6 (79)	32.5 (76)	74.8 (175)	78.7 (181)	75.2 (173)
North Dakota	25	76.0 (19)	60.0 (15)	36.0 (9)	36.0 (9)	84.0 (21)	84.0 (21)	84.0 (21)
Ohio	567	63.7 (361)	45.1 (256)	31.2 (177)	26.8 (152)	48.7 (276)	52.2 (296)	48.7 (276)
Oklahoma	123	95.0 (115)	86.0 (104)	60.3 (73)	40.5 (49)	91.9 (113)	92.7 (114)	91.9 (113)
Oregon	147	59.9 (88)	43.5 (64)	22.6 (33)	25.3 (37)	56.5 (83)	58.5 (86)	55.8 (82)
Pennsylvania	415	74.8 (309)	49.2 (203)	34.1 (141)	34.5 (143)	51.1 (212)	53.6 (222)	51.0 (211)
Puerto Rico	47	52.2 (24)	45.7 (21)	17.4 (8)	23.9 (11)	74.5 (35)	83.0 (39)	72.3 (34)
Rhode Island	41	75.6 (31)	58.5 (24)	39.0 (16)	24.4 (10)	31.7 (13)	41.5 (17)	31.7 (13)
South Carolina	86	90.7 (78)	88.4 (76)	14.0 (12)	9.3 (8)	95.3 (82)	98.8 (84)	95.3 (81)
South Dakota	32	65.6 (21)	50.0 (16)	46.9 (15)	46.9 (15)	62.5 (20)	62.5 (20)	62.5 (20)
Tennessee	233	72.7 (168)	51.9 (120)	37.2 (86)	40.5 (94)	52.8 (123)	54.1 (126)	51.9 (121)
Texas	312	76.6 (239)	68.3 (213)	42.6 (133)	42.9 (134)	69.9 (218)	76.6 (239)	68.9 (215)
Utah	269	52.0 (140)	46.1 (124)	24.6 (66)	28.0 (75)	60.6 (163)	61.3 (165)	59.1 (159)
Vermont	54	79.6 (43)	48.1 (26)	43.4 (23)	37.0 (20)	81.5 (44)	83.3 (45)	81.5 (44)
Virginia	215	57.5 (123)	52.8 (113)	30.8 (66)	29.9 (64)	39.5 (85)	39.1 (84)	38.1 (82)
Washington	279	77.1 (215)	40.9 (114)	33.7 (94)	27.6 (77)	38.7 (108)	40.9 (114)	38.0 (106)
West Virginia	110	64.5 (71)	51.8 (57)	43.6 (48)	48.2 (53)	43.6 (48)	44.5 (49)	43.6 (48)
Wisconsin	278	59.6 (165)	50.9 (141)	30.0 (83)	27.6 (76)	61.5 (171)	65.5 (182)	61.2 (170)
Wyoming	42	83.3 (35)	45.2 (19)	19.0 (8)	19.0 (8)	50.0 (21)	47.6 (20)	47.6 (20)
**Substance use treatment facilities**								
**Overall**	**14,620**	**82.3 (11,978)**	**69.9 (10,192)**	**40.2 (5,857)**	**35.3 (5,145)**	**34.9 (5,092)**	**43.6 (6,356)**	**33.9 (4,945)**
**Facility operation**								
Private for-profit	6,393	77.1 (4,908)	65.1 (4,140)	36.2 (2,302)	31.8 (2,021)	21.2 (1,353)	27.2 (1,735)	20.1 (1,284)
Private nonprofit	6,962	85.6 (5,932)	73.1 (5,085)	42.1 (2,925)	36.7 (2,545)	43.2 (3,006)	54.9 (3,817)	42.2 (2,933)
Public agency or department	1,250	90.7 (1,127)	76.6 (954)	50.4 (627)	46.0 (572)	58.6 (732)	64.2 (803)	58.2 (727)
Service setting***								
Hospital inpatient	1,068	88.3 (941)	84.3 (896)	79.7 (850)	61.1 (651)	36.3 (388)	56.4 (601)	35.6 (379)
Outpatient	12,166	82.1 (9,961)	68.6 (8,327)	35.6 (4,319)	33.4 (4,049)	36.5 (4,441)	41.6 (5,055)	35.5 (4,306)
Residential	3,503	81.8 (2,834)	75.6 (2,639)	61.0 (2,130)	45.8 (1,597)	24.6 (862)	50.2 (1,755)	24.2 (845)
**Jurisdiction**								
Alabama	141	75.7 (106)	63.8 (90)	26.2 (37)	23.4 (33)	21.9 (30)	26.3 (36)	21.2 (29)
Alaska	85	91.8 (78)	72.9 (62)	36.9 (31)	25.0 (21)	56.5 (48)	60.0 (51)	56.5 (48)
Arizona	430	79.0 (338)	59.4 (255)	51.3 (220)	46.4 (199)	25.9 (111)	33.6 (144)	25.2 (108)
Arkansas	127	78.0 (99)	69.3 (88)	30.7 (39)	33.9 (43)	29.9 (38)	32.3 (41)	20.5 (26)
California	1,478	78.1 (1,149)	63.6 (935)	36.7 (539)	31.8 (467)	25.5 (377)	36.1 (532)	24.2 (357)
Colorado	309	82.8 (255)	64.1 (198)	31.2 (96)	34.4 (106)	35.9 (111)	37.2 (115)	34.3 (106)
Connecticut	171	91.1 (153)	77.2 (132)	58.8 (100)	60.0 (102)	44.7 (76)	55.0 (94)	44.7 (76)
Delaware	41	90.2 (37)	82.5 (33)	46.3 (19)	56.1 (23)	41.5 (17)	41.5 (17)	39.0 (16)
District of Columbia	29	72.4 (21)	72.4 (21)	31.0 (9)	31.0 (9)	27.6 (8)	48.3 (14)	27.6 (8)
Florida	625	80.4 (502)	67.3 (419)	43.3 (270)	38.0 (237)	30.9 (193)	37.3 (233)	29.6 (185)
Georgia	285	72.0 (203)	58.5 (166)	29.9 (85)	27.1 (77)	31.6 (90)	47.7 (136)	31.2 (89)
Hawaii	98	86.7 (85)	82.7 (81)	12.2 (12)	11.2 (11)	60.2 (59)	68.4 (67)	60.2 (59)
Idaho	104	72.1 (75)	51.0 (53)	18.4 (19)	25.2 (26)	14.4 (15)	21.2 (22)	13.5 (14)
Illinois	604	74.7 (448)	54.8 (330)	29.4 (177)	23.5 (141)	27.6 (167)	39.0 (235)	26.2 (158)
Indiana	482	84.4 (401)	71.2 (343)	39.1 (188)	44.9 (215)	47.0 (226)	48.3 (232)	43.8 (210)
Iowa	199	90.9 (180)	70.9 (141)	34.7 (69)	25.6 (51)	61.3 (122)	62.8 (125)	60.3 (120)
Kansas	144	75.7 (109)	62.5 (90)	26.4 (38)	18.8 (27)	38.2 (55)	44.4 (64)	38.2 (55)
Kentucky	524	70.5 (368)	63.4 (332)	44.6 (233)	35.4 (185)	9.2 (48)	17.4 (91)	8.2 (43)
Louisiana	188	87.8 (165)	73.3 (137)	50.3 (94)	46.3 (87)	26.1 (49)	42.6 (80)	25.5 (48)
Maine	136	95.5 (128)	71.1 (96)	30.6 (41)	29.9 (40)	41.2 (56)	48.5 (66)	41.2 (56)
Maryland	507	85.2 (426)	65.7 (327)	30.7 (154)	28.1 (141)	20.6 (104)	30.0 (151)	20.3 (102)
Massachusetts	388	94.6 (365)	87.6 (340)	47.9 (186)	43.8 (170)	43.0 (167)	53.1 (206)	42.5 (165)
Michigan	395	75.8 (297)	60.1 (236)	33.4 (131)	27.8 (109)	34.2 (135)	49.1 (194)	33.2 (131)
Minnesota	369	78.3 (289)	62.2 (229)	33.1 (121)	25.4 (93)	19.8 (73)	34.7 (128)	19.8 (73)
Mississippi	99	78.8 (78)	66.7 (66)	32.3 (32)	28.3 (28)	30.3 (30)	41.4 (41)	30.3 (30)
Missouri	246	83.7 (205)	73.0 (178)	53.1 (130)	51.4 (126)	38.2 (94)	48.4 (119)	38.2 (94)
Montana	85	81.2 (69)	75.0 (63)	21.4 (18)	20.2 (17)	39.3 (33)	36.9 (31)	36.9 (31)
Nebraska	103	86.4 (89)	59.2 (61)	29.1 (30)	31.1 (32)	38.8 (40)	56.3 (58)	38.8 (40)
Nevada	116	74.8 (86)	73.0 (84)	40.9 (47)	40.9 (47)	28.4 (33)	31.0 (36)	25.0 (29)
New Hampshire	91	95.6 (87)	80.2 (73)	54.9 (50)	54.9 (50)	44.4 (40)	44.0 (40)	41.1 (37)
New Jersey	374	86.9 (325)	76.6 (285)	39.6 (147)	33.5 (124)	21.7 (81)	31.8 (119)	20.3 (76)
New Mexico	151	80.8 (122)	56.3 (85)	35.1 (53)	35.1 (53)	35.8 (54)	48.3 (73)	35.1 (53)
New York	759	96.4 (730)	93.5 (705)	75.8 (574)	64.2 (486)	76.8 (582)	84.0 (637)	76.0 (576)
North Carolina	514	80.9 (415)	74.1 (380)	45.0 (231)	39.6 (203)	52.7 (271)	57.9 (296)	51.1 (261)
North Dakota	59	93.2 (55)	69.5 (41)	40.7 (24)	37.3 (22)	37.3 (22)	55.9 (33)	32.2 (19)
Ohio	677	81.7 (552)	70.3 (475)	46.4 (314)	39.4 (267)	21.6 (146)	29.6 (200)	21.6 (146)
Oklahoma	165	95.7 (157)	90.3 (149)	42.7 (70)	24.5 (40)	87.9 (145)	90.9 (150)	87.9 (145)
Oregon	213	93.4 (199)	81.5 (172)	34.8 (73)	30.0 (63)	54.0 (115)	63.8 (136)	53.1 (113)
Pennsylvania	553	83.3 (459)	65.9 (363)	36.2 (200)	29.0 (160)	22.8 (126)	35.4 (196)	22.4 (124)
Puerto Rico	62	45.2 (28)	56.5 (35)	11.3 (7)	14.5 (9)	41.9 (26)	87.1 (54)	41.9 (26)
Rhode Island	60	80.0 (48)	76.7 (46)	33.3 (20)	18.3 (11)	38.3 (23)	41.7 (25)	36.7 (22)
South Carolina	105	74.3 (78)	72.4 (76)	22.9 (24)	11.4 (12)	41.0 (43)	42.3 (44)	38.5 (40)
South Dakota	46	87.0 (40)	76.1 (35)	39.1 (18)	26.1 (12)	41.3 (19)	54.3 (25)	41.3 (19)
Tennessee	319	83.3 (265)	70.2 (224)	44.2 (141)	35.1 (112)	23.2 (74)	35.5 (113)	22.6 (72)
Texas	497	86.1 (426)	82.9 (411)	29.4 (146)	26.0 (129)	43.1 (214)	54.3 (270)	42.3 (210)
Utah	287	79.4 (224)	72.7 (208)	51.8 (147)	46.7 (133)	36.6 (105)	38.7 (111)	35.5 (102)
Vermont	51	96.1 (49)	88.2 (45)	56.9 (29)	52.9 (27)	66.7 (34)	70.6 (36)	66.7 (34)
Virginia	339	72.5 (242)	62.8 (213)	43.2 (146)	35.5 (120)	23.7 (80)	27.5 (93)	23.4 (79)
Washington	350	92.3 (322)	76.0 (266)	31.6 (110)	25.0 (87)	30.9 (108)	38.6 (135)	30.6 (107)
West Virginia	143	82.5 (118)	72.7 (104)	51.0 (73)	42.7 (61)	30.8 (44)	36.4 (52)	30.8 (44)
Wisconsin	236	76.6 (180)	70.2 (165)	30.9 (73)	31.8 (75)	46.8 (110)	53.6 (126)	46.8 (110)
Wyoming	56	91.1 (51)	83.9 (47)	38.2 (21)	47.3 (26)	42.9 (24)	53.6 (30)	41.1 (23)

* Estimates for mental health and substance use treatment facilities are not mutually exclusive because they each include facilities offering both substance use and mental health treatment services. Caution should be exercised when comparing and interpreting findings across facility types.

^†^ Data for tobacco use screening were missing for 30 mental health treatment facilities (ranging by jurisdiction from zero to three facilities) and 69 substance use treatment facilities (ranging by jurisdiction from zero to seven facilities).

^§^ Data for tobacco cessation counseling were missing for 35 mental health treatment facilities (ranging by jurisdiction from zero to four facilities) and 48 substance use treatment facilities (ranging by jurisdiction from zero to nine facilities).

^¶^ Data for nicotine replacement therapy were missing for 40 mental health treatment facilities (ranging by jurisdiction from zero to five facilities) and 56 substance use treatment facilities (ranging by jurisdiction from zero to eight facilities).

** Data for non-nicotine cessation medications were missing for 41 mental health treatment facilities (ranging by jurisdiction from zero to five facilities) and 65 substance use treatment facilities (ranging by jurisdiction from zero to 10 facilities).

^††^ Data for smoke-free policies were missing for nine mental health treatment facilities (ranging by jurisdiction from zero to two facilities) and 19 substance use treatment facilities (ranging by jurisdiction from zero to four facilities).

^§§^ Data for vape-free policies were missing for 17 mental health treatment facilities (ranging by jurisdiction from zero to five facilities) and 28 substance use treatment facilities (ranging by jurisdiction from zero to five facilities).

^¶¶^ Data for tobacco-free policies were missing for 17 mental health treatment facilities (ranging by jurisdiction from zero to five facilities) and 31 substance use treatment facilities (ranging by jurisdiction from zero to five facilities).

*** Service setting categories are not mutually exclusive.

The percentage of facilities offering cessation counseling varied by jurisdiction; ranging, among mental health facilities, from 30.3% in Idaho to 88.4% in South Carolina and, among substance use facilities, from 51.0% in Idaho to 93.5% in New York. NRT provision also varied by jurisdiction, ranging from 14.0% in South Carolina to 65.0% in New Hampshire among mental health facilities, and from 11.3% in Puerto Rico to 75.8% in New York among substance use facilities.

### Tobacco-Free, Smoke-Free, and Vape-Free Policies

Tobacco-free policies were reported by 53.9% of mental health and 33.9% of substance use facilities. More vape-free policies (57.9% of mental health and 43.6% of substance use facilities) were reported than were smoke-free policies (54.6% of mental health and 34.9% of substance use facilities). The highest percentages of vape-free and smoke-free policies were reported by public agency–operated facilities (when stratified by facility operation), irrespective of facility type. Among mental health facilities, the highest percentages of vape-free and smoke-free policies were reported by hospital inpatient facilities (when stratified by service setting). Among substance use facilities, hospital inpatient facilities had the highest percentage of vape-free policies; outpatient and hospital inpatient facilities reported similar percentages of smoke-free policies.

Smoke-free policies varied by jurisdiction. Among mental health facilities, prevalences ranged from 29.9% in Nevada to 95.3% in South Carolina. Among substance use facilities, prevalences ranged from 9.2% in Kentucky to 87.9% in Oklahoma. The percentage of facilities with vape-free policies also varied by jurisdiction; ranging, among mental health facilities, from 36.4% in Nevada to 98.8% in South Carolina and, among substance use facilities, from 17.4% in Kentucky to 90.9% in Oklahoma.

### Tobacco Cessation Services Offered Among Facilities with Tobacco-Free Policies

Nationally, among facilities with tobacco-free policies, 64.4% of mental health and 81.8% of substance use facilities offered at least one tobacco cessation service (Supplementary Table, https://stacks.cdc.gov/view/cdc/177493#tabs). In 23 jurisdictions, more than 70% of mental health facilities with a tobacco-free policy offered at least one cessation service (Figure). In 46 jurisdictions, more than 70% of substance use facilities with a tobacco-free policy offered at least one cessation service.

**FIGURE:**
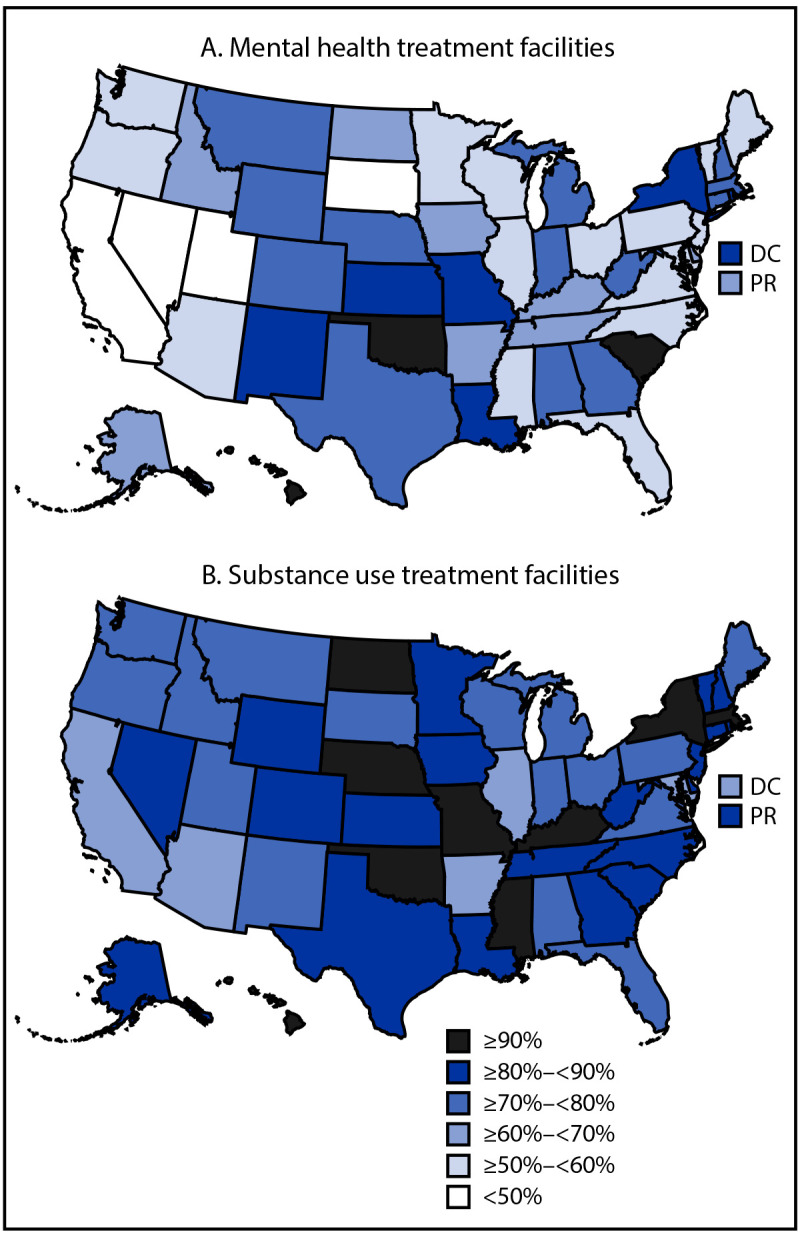
Percentage of behavioral health treatment facilities with a tobacco-free policy that offer at least one tobacco cessation service, by facility type — National Substance Use and Mental Health Services Survey, 52 jurisdictions, United States, 2023 **Abbreviations:** DC = District of Columbia; PR = Puerto Rico.

## Discussion

These findings suggest progress and continued opportunity for improvement in the availability of tobacco cessation supports in behavioral health treatment settings. For example, in 2016, 48.9% of mental health and 64.0% of substance use facilities reported tobacco-use screening, and 37.6% of mental health and 47.4% of substance use facilities reported offering counseling (*8*). In 2023, 69.2% of mental health and 82.3% of substance use facilities reported offering screening, and 53.1% of mental health and 69.9% of substance use facilities reported offering counseling. Although recent data might not be directly comparable to those from earlier years because of survey design changes in 2021,** the current findings suggest increases in availability of cessation supports. This progress is likely related to coordinated public health efforts. For example, since 2018, SAMHSA has funded a National Center of Excellence for Tobacco-Free Recovery to increase tobacco cessation supports in behavioral health care.^††^ In 2020, CDC’s National Tobacco Control Program^§§^ began requiring state health departments to address tobacco use among persons with behavioral health conditions.^¶¶^ Several jurisdictions have focused on treatment settings as part of this work.***

South Carolina, for example, developed interagency partnerships to support implementation of initiatives to reduce tobacco use among persons with behavioral health conditions.^†††^ As a result, 100% of community mental health centers and 59% of local Alcohol and Drug Abuse Commissions in South Carolina have adopted tobacco-free policies. In addition, South Carolina has observed an increase in the number of providers diagnosing tobacco use disorder and in quitline callers enrolled in cessation counseling tailored for persons with behavioral health conditions.

In Indiana, tobacco-free recovery grants have focused on system-level strategies.^§§§^ Leveraging multisector relationships, Indiana established learning collaboratives focused on implementing tobacco-free policies and tobacco treatment and referral protocols in behavioral health settings. As a result of these efforts, more than 80% of state-funded behavioral health agencies reported routinely incorporating tobacco dependence treatment into their treatment planning processes in 2023, an increase from approximately 40% in 2017.

Opportunities remain to further expand the availability of cessation supports in behavioral health treatment settings, particularly given that fewer than one half of treatment facilities offered cessation pharmacotherapy in 2023. Continued efforts to educate behavioral health professionals about cessation treatment strategies and the benefits of smoking cessation for behavioral health outcomes might be warranted. In addition, health systems–based strategies, such as implementation of treatment protocols and clinical workflows, can help reduce the strain on clinical staff and systematize screening and treatment (*4*). Legislative and regulatory strategies are also being used by states, including laws or regulations mandating tobacco-free policies^¶¶¶^ or requiring the availability of tobacco cessation treatment in mental health and substance use facilities.****

Many persons with behavioral health conditions want to quit smoking and can quit (*3**,**4*). Quitting smoking is associated with positive mental health outcomes, including decreased anxiety, depressive symptoms, and stress, as well as improved substance use recovery outcomes (*3*–*7*). Quitting does not interfere with behavioral health treatment or impede substance use recovery (*3*–*5*). Evidence-based treatments can help persons with behavioral health conditions quit, although some studies suggest that these persons might require longer duration or more intensive treatments (*4*). Research is needed to better understand how to maximize the impact and effectiveness of cessation treatments tailored to this group.

### Limitations

The findings in this report are subject to at least three limitations. First, responses were self-reported and subject to reporting bias. Second, the analysis did not include nonresponse adjustments to minimize nonresponse bias for facilities that did not respond to the survey. Finally, the survey did not assess delivery or use of cessation services or implementation or enforcement of tobacco-free policies.

### Implications for Public Health Practice

Supporting tobacco cessation in behavioral health treatment settings is an important component of a comprehensive approach to reducing tobacco use and related health outcomes among persons with behavioral health conditions. This analysis identified substantial gaps in the availability of tobacco cessation treatments and tobacco-free policies at behavioral health treatment facilities. Increasing implementation of tobacco-free policies and integrating tobacco cessation treatment into behavioral health care could support cessation and help decrease tobacco-related disease and might improve behavioral health outcomes.

## Footnotes

* Commercial tobacco refers to tobacco products that are made and sold by companies. This definition does not include traditional tobacco used by some Indigenous groups for religious or ceremonial purposes. In this report, the term “tobacco” refers to commercial tobacco products including combustible products and e-cigarettes.

†
https://www.samhsa.gov/data/

§ Service setting categories are not mutually exclusive; partial hospitalization or day treatment facilities provide only partial day services to ambulatory clients, typically in sessions of ≥3 hours, on a regular schedule. https://www.samhsa.gov/data/system/files/media-puf-file/N-SUMHSS-2023-DS0001-info-codebook_v1.pdf

¶ 45 C.F.R. part 46.102(l)(2), 21 C.F.R. part 56; 42 U.S.C. Sect. 241(d); 5 U.S.C. Sect. 552a; 44 U.S.C. Sect. 3501 et seq.

**
https://www.samhsa.gov/data/data-we-collect/n-sumhss-national-substance-use-and-mental-health-services-survey

†† SAMHSA’s National Center of Excellence for Tobacco-Free Recovery provides technical assistance, training, and resources to promote integration of tobacco treatment and adoption of tobacco-free policies into behavioral health treatment settings. As part of this work, the center convenes State Leadership Academies for Tobacco-Free Recovery to develop and implement statewide action plans to address tobacco use in behavioral health population groups. https://www.samhsa.gov/resource/tta/national-center-excellence-tobacco-free-recovery-coe-tfr

§§ CDC’s National Tobacco Control Program provides funding and technical assistance to state and territorial tobacco control programs. https://www.cdc.gov/tobacco/php/tobacco-control-programs/index.html

¶¶ Tobacco control programs subject to this requirement were those in states where the proportion of behavioral health treatment facilities offering tobacco use screening and smoke-free policies fell below the 2016 national average based on data from the National Survey of Substance Abuse Treatment Services and the National Mental Health Services Survey.

*** Based on the internal National Tobacco Control Program Evaluation Report, years 1 and 2, National Tobacco Control Program Awards Management Platform.

††† Based on the internal National Tobacco Control Program Impact Statement: South Carolina.

§§§ Based on the internal National Tobacco Control Program Impact Statement: Indiana.

¶¶¶ As of July 2024, 18 states and DC had laws or regulations mandating tobacco-free policies for most mental health facilities and 19 states had similar requirements for most substance use facilities. https://www.publichealthlawcenter.org/sites/default/files/resources/Tobacco-Free-State-Policies-Mental-Health-Substance-Use-Facilities.pdf

**** As of September 2024, seven states required the availability of tobacco cessation treatment in mental health facilities and nine states in substance use facilities. https://www.publichealthlawcenter.org/sites/default/files/resources/Tobacco-Cessation-Treatment-State-Policies.pdf
